# Physical and chemical properties, hygienic quality and fatty acid profile in milk of lactating Lacaune dairy sheep

**DOI:** 10.5194/aab-67-37-2024

**Published:** 2024-02-02

**Authors:** Zvonko Antunović, Boro Mioč, Željka Klir Šalavardić, Ivan Širić, Valentino Držaić, Nataša Mikulec, Adela Krivohlavek, Josip Novoselec

**Affiliations:** 1 Department of Animal Production and Biotechnology, Faculty of Agrobiotechnical Sciences Osijek, University of J. J. Strossmayer in Osijek, V. Preloga 1, 31000 Osijek, Croatia; 2 Department of Animal Science and Technology, Faculty of Agriculture, University of Zagreb, Svetošimunska cesta 25, 10000 Zagreb, Croatia; 3 Department of Dairy Science, Faculty of Agriculture, University of Zagreb, Svetošimunska cesta 25, 10000 Zagreb, Croatia; 4 Andrija Štampar Teaching Institute of Public Health, Mirogojska 16, 10000 Zagreb, Croatia

## Abstract

In recent years, there has been globally increasing interest in dairy sheep breeding, including Lacaune sheep, which is supported by a high demand for sheep's milk on the market. This paper elaborates on the influence of a sheep's lactation stage on the physical and chemical properties, hygienic quality and content of fatty acids in milk produced by Lacaune sheep kept in intensive breeding. The research was conducted on 30 Lacaune sheep, which were tested in the early (60th day), middle (120th day) and late (180th day) stages of lactation. Density, freezing point and titration acidity were determined by applying the infrared spectrometry method, and indicators of the hygienic quality of milk, such as somatic cell count (SCC), were determined by the fluoro-opto-electronic method, and the total count of aerobic mesophilic bacteria (CFU) was determined by the flow cytometry method. The fatty acid profile of feed and milk was obtained by gas–liquid chromatography. Depending on the stage of lactation, results referring to the chemical composition of Lacaune sheep's milk showed a significant increase in the content of fat, protein, total dry matter and casein together with a significant decrease in the content of lactose in the late stage of lactation. There was also a significant increase confirmed for the concentration of urea and the freezing point in milk along with the lactation progress. Depending on the stage of lactation, milk yield in Lacaune sheep significantly decreased as lactation progressed. Analysis of the fatty acid composition in milk of Lacaune sheep proved a significant decrease in the concentrations of C4 : 0, C6 : 0, C11 : 0, C12 : 0, C13 : 0, C15 : 0, C17 : 1, C18 : 2n6 and C18 : 3n6 as well as the n6 concentrations and the n6 / n3 ratio. The opposite trend was observed for concentrations of C10 : 0, C14 : 1, C16 : 0, C16 : 1, C18 : 0, C20 : 2, C18 : 3n3, C20 : 3n6, C20 : 5n3 and C22 : 6n3 as well as for the n3 concentrations. When compared to the early lactation stage, the C18 : 3n6 and n6 concentrations were significantly lower in the late lactation stage, while the C20 : 2 and C20 : 5n3 concentrations were significantly lower in the middle lactation stage when compared to the late lactation stage. There were many significant positive and negative correlations determined between the researched properties of milk. The research results obtained with Lacaune sheep's milk can be compared to the results of other studies, except for the lower content of milk fat. This confirms the good adaptability of Lacaune sheep to different breeding conditions and the necessity to provide sheep with quality pastures for grazing.

## 1 Introduction

There is a high demand for sheep's milk on the global market, which affects the demand for high-yielding dairy sheep breeds (Li et al., 2022). The French Lacaune is one such dairy sheep breed being exported worldwide (Barillet et al., 2001) and is used in pure breeding or crossbreeding in order to improve the milk yield of domestic sheep populations (Antunović et al., 2022a, b; Jimenez et al., 2020; Panayotov et al., 2018a). The price of sheep's milk is 2 to 3 times higher than cow's milk, which significantly affects the production outcomes (Legarra et al., 2007). The global demand for sheep's milk is supported by comprehensive research into the quality of such milk. Flis and Molik (2021) pointed out that sheep's milk was an important source of bioactive ingredients that have health-promoting functions for the human body.

Over the last decade, the focus has been on research into the chemical composition, hygienic quality and fatty acid profile of milk from various dairy sheep breeds (Hassoun et al., 2021; Antunović et al., 2017; Alizadeh et al., 2017; Sinanoglou et al., 2015; Makovicky et al., 2013; Mierlita et al., 2011; Park et al., 2007). Dairy sheep production in Croatia is based on breeding of autochthonous sheep breeds, mostly in the Mediterranean part of the country. However, the recently growing demand for sheep's milk caused greater import of smaller herds of Lacaune sheep to the continental part of the country, where well-developed intensive agriculture provides cereals which serve as a basis for Lacaune sheep feed. Most sheep's milk is processed into cheeses, which are also in high demand on the market. The first preliminary results of Lacaune sheep breeding in Croatia indicate their good adaptation to the existing conditions (Antunović et al., 2022a, b, c, d). Studies conducted on Lacaune sheep in countries in Croatia's vicinity (Italy, Hungary, Slovakia, Czech Republic, Bulgaria) as well as in more distant countries (Spain, Estonia, USA, Brazil) report similar conclusions. Many studies showed that the chemical content, hygienic quality and fatty acid profile of sheep's milk vary significantly, which is also the case in milk of Lacaune sheep. There are several milk-affecting factors, such as feeding regime, genotype, physiological stage, parity, locality, season and some others (Tsiplakou et al., 2008; de La Fuente et al., 2009; Soják et al., 2013; González-García et al., 2015; Panayotov et al., 2019; Correddu et al., 2019). Nudda et al. (2020) stated that different sheep feeding regimes could significantly influence the concentration of milk fat and improve the fatty acid profile of yielded milk. Chilliard et al. (2007) concluded that feeding of sheep on pastures influenced the decreased saturated fatty acid (SFA) content and the increased favourable fatty acid content (oleic acid, α-linoleic acid and conjugated linoleic acid (CLA)) in milk. In addition, Conte et al. (2022) confirmed that, in addition to the feeding regime, there was a significant influence of genotype, so the sheep breed should be considered an important criterion in the research into fatty acids in sheep's milk. The influence of the lactation stage on the physical and chemical properties, hygienic quality as well as content and profile of fatty acids in milk has not yet been thoroughly investigated in Lacaune sheep kept under intensive farming conditions. Therefore, the aim of this research is to investigate the influence of the lactation stage on the physical and chemical properties, on hygienic quality and on the content of fatty acids in milk of Lacaune sheep kept in intensive farming.

## 2 Material and methods

### 2.1 Experimental design and selection of animals

The research was conducted on Lacaune sheep kept at the Orkić family farm in Gundinci, Croatia (latitude: 45.155; longitude: 18.492). The experimental animals were selected from a flock of 200 Lacaune sheep. The key selection criteria were lactation stage, age, order of lactation and one delivered lamb. The sheep were selected immediately after weaning of lambs. The experiment involved 30 sheep that were monitored in the early (60th day), middle (120th day) and late (180th day) stages of lactation. The average sheep body weight was 61 kg and the body condition score 3.05. All the selected sheep were healthy and in good physical condition.

### 2.2 Sheep nutrition and feed analysis

The sheep were fed pelleted feed with 15 % crude protein with an amount of 1.00 kg d${}^{-1}$, a mixture of cereals (one-third oat and two-thirds barley) with an amount of 600 g d${}^{-1}$ and alfalfa hay ad libitum. They were also given animal salt and water ad libitum. Feed samples (feed mixture, cereal mixture and hay) were dried and ground into a fine powder by using a heavy-metal-free ultra-centrifugal mill (Retsch ZM 200) or knife mill (GM 200). The composition of the feed was determined by using standard methods (AOAC, 2006). The chemical compositions of the feed are presented in Table 1.

**Table 1:** Chemical composition of Lacaune sheep feed.

Parameter	Feed		
(g kg${}^{-1}$ DM)	Feed mixture	Cereal mixture	Hay
DM	917.20	910.10	914.60
Crude proteins	149.9	139.1	136.7
Crude fibre	51.1	44.1	311.0
Crude ash	50.6	20.1	66.5
Ether extract	26.9	28.0	10.1
NDF	36.56	40.06	65.06
ADF	7.1	4.93	40.8
ADL	2.22	1.29	10.25
Fatty acid (g 100 g${}^{-1}$ fat)			
C6 : 0	0.32	0.84	0.32
C8 : 0	0.18	0.35	0.31
C10 : 0	0.07	0.09	0.31
C12 : 0	0.14	0.15	1.04
C14 : 0	0.99	0.86	2.69
C15 : 0	0.12	0.12	0.79
C15 : 1n5	0.55	1.06	0.11
C16 : 0	21.59	38.18	39.77
C16 : 1n7	0.59	0.43	1.04
C17 : 0	0.19	0.18	1.01
C17 : 1n7	0.08	0.07	0.24
C18 : 0	3.53	3.66	9.12
C18 : 1n9	0.20	0.35	0.37
C18 : 1n9	29.98	33.11	15.22
C18 : 2n6	29.72	7.35	4.93
C18 : 3n3	0.60	0.13	0.65
C20 : 0	0.45	0.58	1.70
C20 : 1n9	1.48	1.53	1.08
C20 : 2n6	0.12	0.16	0.17
C21 : 0	0.09	0.12	0.32
C22 : 0	0.35	0.30	2.26
C22 : 2n6	0.50	0.66	0.45
C23 : 0	0.50	0.78	2.00
C24 : 0	0.29	0.35	2.80
C24 : 1n9	0.14	0.07	0.07
C18 : 1n7	0.96	0.94	0.89
C18 : 4n3	0.13	0.19	0.54
C20 : 1n11	0.15	< 0.05	0.13
C22 : 1n11	1.78	0.77	1.14
C18 : 2n6	0.10	0.11	0.36
C18 : 3n3	< 0.05	0.06	0.06
SFA	28.80	46.58	64.70
MUFA	35.99	38.33	20.36
PUFA	31.56	8.65	7.66
n3	1.12	0.37	1.25
n6	30.44	8.27	6.41
n9	31.87	35.06	16.75

DM – dry matter; NDF – neutral detergent fibre; ADF – acid detergent fibre; ADL – acid detergent lignin; SFA – saturated fatty acids; MUFA – monounsaturated fatty acids; PUFA – polyunsaturated fatty acids; n3 – total n3 fatty acids; n6 – total n6 fatty acids.

After hydrolysing the feed sample in a hydrolysis device (Hydrotherm Hydrolysis System V02, Gerhardt GmbH & Co. KG, Königswinter, Germany), which is carried out in 4 N HCl, the samples were dried at 60 ${}^{\circ }$C and subjected to extraction in an extraction device (Soxtherm SOX SE-416, Gerhardt GmbH & Co. KG, Königswinter, Germany) by running the programme (extraction at 150 ${}^{\circ }$C, reduction interval 0.40 min, reduction pulse 2 s, extraction 30 min (boiling), steaming A 5× interval, washing time 50 min (extraction), steaming B 3× interval). After the extraction, the sample was dried in a dryer (UN 55, Memmert GmbH + Co. KG, Schwabach, Germany) for ca. 30 min at 103 ${}^{\circ }$C in order to remove the remaining solvent. Afterwards, the sample was cooled down to room temperature and weighed on a balance with an accuracy of 0.1 mg (AG204, Mettler Toledo Columbus, Ohio, USA), and then the mass fraction of fat (%) was calculated from the sample.

The base–acid derivatization procedure was implemented by mixing a homogenized sample (25 mg) with 2 mL of a 0.2 M solution of sodium methoxide in methanol (NaOH: p.a., Grammol, Zagreb, Croatia; methanol: HPLC grade, Supelco, Darmstadt, Germany). Then the sample was heated on a thermal block (Thermoreactor CR3000, WTW, DG Apeldoorn, Netherlands) (T≈100 ${}^{\circ }$C) with occasional shaking (every 2–3 min) until the emulsion disappeared. The sample was removed from the thermal block, cooled well under running water, and mixed with two to three drops of phenolphthalein solution. While stirring constantly, a sample was mixed with a 1 M solution of sulfuric acid (95 %–97 %, EMSURE, ISO p.a. Supelco, Darmstadt, Germany) in methanol until the solution changed colour and became clear. After that, the volume of the solution was measured and the content was heated on a thermal block (T≈100 ${}^{\circ }$C) for about 10 min by occasional shaking of the test tube (every 2–3 min). After heating, the sample was cooled well under running water, and then 3 mL of saturated sodium chloride solution was added, mixed well and left to form a white residue of NaCl. Then the sample was mixed with 2 mL of isooctane (≥99.8 % GC for gas chromatography with an electron capture detector and a flame ionization detector; SupraSolv^®^ Supelco, Darmstadt, Germany) and left to rest for a few minutes for the phases to separate when washed with saturated sodium chloride solution. The upper, clear isooctane layer containing methyl esters of fatty acids was transferred with a Pasteur pipette into an injection vial previously marked with the sample number. Such a prepared sample was analysed in a gas chromatograph equipped with a flame ionization detector (GC 2010 Plus GC-FID, Shimatzu, Kyoto, Japan) in a 60 m × 0.25 mm × 0.25 µm DB-23 column (Agilent, Santa Clara, USA). The temperature programme started at 60 ${}^{\circ }$C for 1 min, increased to 215 ${}^{\circ }$C, and was then maintained for 30 min for a total of 54 min with gas flows of 30, 40 and 400 mL min${}^{-1}$ for makeup gases N${}_{2}$ or air, H${}_{2}$, and air, respectively. The chemical composition of the feed, the determination of the NDF (neutral detergent fibre), ADF (acid detergent fibre) and ADL (acid detergent lignin), and the application of the methods are described in the study by Antunović et al. (2021).

### 2.3 Sampling and analysis of milk

The amount of milk was determined by analysing the morning milk yield of sheep in the early, middle and late stages of lactation. Samples for physical and chemical analyses were collected in 200 mL bottles. Samples for determining the hygienic quality of milk were collected in sterile 30 mL bottles. Samples were kept in a portable refrigerator (+4 ${}^{\circ }$C) for 3 to 5 h and delivered to the Dairy Institute of the Faculty of Agriculture, University of Zagreb, Croatia. The contents of milk fat, lactose, protein, dry matter, casein, citrate, galactose, glucose, free fatty acid, urea, density, freezing point and titration acidity in milk were determined by the infrared spectrometry method (HRN ISO 9622:2017) on the MilkoScan FT3 device (Foss Electric, Denmark), which operates by Fourier transformation of the infrared spectrum. The hygienic quality of the milk was determined by measuring the somatic cell count (SCC) and the total microorganism count. The SCC in the milk was determined by the fluoro-opto-electronic method (HRN EN ISO 13366-3:2008) on the Fossomatic Minor device (Foss Electric, Denmark), and the total count of the aerobic mesophilic bacteria (CFU) was measured by the flow cytometry method (ISO 21187:2021, HRN ISO 4833:2013) on the Bactoscan FC device (Foss Electric, Denmark) using the internal correction factor for the individual bacterial count (IBC). The obtained data were log-transformed to normalize the distribution. All the analyses were repeated twice. Determination of the composition of milk fat by gas–liquid chromatography was carried out by preparing methyl esters of fatty acids according to the standard HRN ISO 15884 (Milk fat – Preparation of methyl esters of fatty acids) and by instrumentally analysing a gas–liquid chromatograph according to the standard HRN ISO 15885 (Milk fat – Determination of the composition of milk fat by gas liquid chromatography).

Extraction of lipids, esterification of fatty acids into methyl esters and analysis on a gas–liquid chromatograph with FID (Shimadzu CG – 2010 Plus) were performed in order to determine the composition of fatty acids. A chromatograph column IntertCap Pure Wax (0.25 mm i.d. × 30 m, df = 0.25 µm) and FID were used for fatty acid analysis. By comparing the retention times and peaks of the analysed sample to the standard (FAME MIX), the proportion of each individual fatty acid in the sample was quantified. Sheep milk samples were stored at +4 ${}^{\circ }$C until they were subjected to analysis. The prepared sample mixed with the solvent is injected into the gas chromatograph injector through which the carrier gas flows, which is designed to move the sample substances through the column to separate it into components. Each component passes by an electronic detector that identifies it and prints a chromatographic peak on the chromatogram. Prior to experiment sample analysis, quality controls were performed by analysing blank samples, reference materials and the standard FAME MIX 37 (Supelco, USA).

A method for the fatty acid identification was previously created according to the given parameters (i) injector temperature 250 ${}^{\circ }$C, (ii) column temperature 50 ${}^{\circ }$C (5 min) → 5 ${}^{\circ }$C min${}^{-1}$ → 260 ${}^{\circ }$C (30 min) and (iii) carrier gas helium. Qualitative identification of fatty acids was obtained by comparing the retention times (RTs) of the recorded chromatograms of the samples and the standard FAME MIX. The GC Solution software automatically provided the results of all the marked peaks as displayed on the chromatogram, and the quantification of shares of individual fatty acids in the sample was done by comparing the sample peaks to FAME MIX.

### 2.4 Statistical analyses

The obtained research results were processed by the MEANS procedure, while the influence of the lactation stages was analysed by the generalized linear model (PROC GLM) procedure and processed by the SAS 9.4^®^. The means were compared using the Tukey test, and the differences between the groups were declared significant at P<0.05. Values of SCC and CFU were logarithmically converted to a linear score with the aim of approximating a normal distribution. Correlations between indicators of ewes' milk were evaluated by Pearson's correlation with the CORR procedure. The correlations were declared significant if P<0.05.

## 3 Results

Table 2 shows descriptors for physical and chemical properties and hygienic indicators of sheep's milk, together with the daily milk yield of Lacaune sheep.

**Table 2:** Descriptive statistics of the physical and chemical properties as well as hygienic indicators of milk and the daily milk yield of Lacaune sheep.

Parameter, g 100 g${}^{-1}$	Mean	SD	Minimum	Maximum
Fat	5.53	1.18	3.69	9.46
Protein	5.90	0.80	4.71	8.51
Lactose	4.51	0.39	3.14	5.14
TS	17.40	1.80	13.95	24.13
SNF	11.29	0.59	9.81	13.19
Casein	4.60	0.60	3.72	6.52
Citric	0.12	0.04	0.03	0.24
Density, g L${}^{-1}$	1036.35	1.70	1031.70	1042.40
FFA, m eq.	0.46	0.16	0.05	0.71
FP, m ${}^{\circ }$C	-575.06	16.49	-622.60	-541.70
Galactose	0.16	0.09	0.01	0.36
Glucose	0.16	0.14	0.00	0.54
TA, ${}^{\circ }$ TH	22.78	1.86	18.80	27.10
Urea, mg dL${}^{-1}$	26.43	14.32	5.00	60.00
logSCC	5.51	0.59	4.62	6.68
logCFU	6.123	0.50	4.84	7.00
Milk yield, g	1399.08	544.54	331.52	2558.92

SD – standard deviation; TS – total solids; SNF – solid non-fat; FFA – free fatty acids; FP – freezing point; TA – titratable acidity; SCC – somatic cell count; CFU – total number of microorganisms.

The average values determined for most of the physical and chemical properties and hygienic indicators of milk did not vary significantly, except for the higher values of standard deviation referring to freezing point (FP) and milk yield.

**Table 3:** Influence of lactation stages on physical and chemical properties, hygienic indicators of milk and the daily milk yield of Lacaune sheep.

Parameter, g 100 g${}^{-1}$	Stage of lactation			SEM	P value
	I	II	III		
Fat	4.84${}^{b}$	5.39${}^{b}$	6.36${}^{a}$	0.125	<0.001
Protein	5.52${}^{b}$	5.51${}^{b}$	6.69${}^{a}$	0.085	<0.001
Lactose	4.72${}^{a}$	4.73${}^{a}$	4.10${}^{b}$	0.040	<0.001
TS	16.48${}^{b}$	17.05${}^{b}$	18.66${}^{a}$	0.190	<0.001
SNF	11.16	11.23	11.48	0.062	0.082
Casein	4.30${}^{b}$	4.34${}^{b}$	5.17${}^{a}$	0.063	<0.001
Citric	0.12	0.13	0.11	0.003	0.050
Density, g L${}^{-1}$	1036.98	1035.97	1036.11	0.180	0.050
FFA, m eq.	0.50${}^{b}$	0.58${}^{a}$	0.29${}^{c}$	0.017	<0.001
FP, m ${}^{\circ }$C	$-561.40^{b}$	$-579.40^{a}$	$-584.37^{a}$	1.738	<0.001
Galactose	0.18	0.17	0.14	0.009	0.328
Glucose	0.16	0.20	0.123	0.016	0.134
TA, ${}^{\circ }$ TH	21.98${}^{b}$	22.37${}^{b}$	23.99${}^{a}$	0.196	<0.001
Urea, mg dL${}^{-1}$	16.17${}^{c}$	36.13${}^{a}$	27.00${}^{b}$	1.509	<0.001
logSCC	5.44	5.43	5.67	0.063	0.185
logCFU	5.92	6.24	6.20	0.53	0.050
Milk yield, g	1772.60${}^{a}$	1526.37${}^{a}$	910.98${}^{b}$	58.381	<0.001

SEM – standard error of the mean; ${}^{a,b,c}$ – means with different superscript letters differ significantly (p<0.05); TS – total solids; SNF – solid non-fat; FFA – free fatty acids; FP – freezing point; TA – titratable acidity; SCC – somatic cell count; CFU – total number of microorganisms.

Referring to the lactation stages (Table 3), results of chemical composition of Lacaune sheep's milk confirmed that there was a significant increase in the content of fat, protein, total dry matter and casein in the late lactation stage compared to the other two stages. When compared to the first (early) lactation stage, there was also an increase in the above-mentioned milk indicators observed in the second (middle) lactation stage but without significant differences. However, sheep's milk sampled during the late lactation stage had a significantly reduced content of lactose compared to milk sampled during the other two stages. Along with the lactation progress, milk sampled during all three stages of lactation had significantly increased concentrations of urea and FP. Furthermore, there was also a significant increase and then a significant decrease in FFA content determined as lactation progressed to the late stage. Milk yield in Lacaune sheep significantly decreased as lactation progressed. Hygienic indicators of milk (SSC and CFU) did not differ significantly during different stages of lactation, although those indicators increased along with the lactation progress.

**Table 4:** Descriptive statistics of the fatty acid content and fatty acid groups in Lacaune ewes' milk (g 100 g${}^{-1}$ fat).

Parameter	Mean	SD	Minimum	Maximum
C4 : 0	3.46	1.256	1.676	8.18
C6 : 0	2.71	0.93	1.19	6.38
C8 : 0	2.51	0.95	0.67	5.77
C10 : 0	8.93	3.59	1.44	16.63
C11 : 0	0.57	0.31	0.00	1.06
C12 : 0	6.43	1.98	1.60	11.47
C13 : 0	0.55	0.29	0.00	1.20
C14 : 0	12.14	1.72	6.03	15.55
C14 : 1	0.44	0.27	0.12	1.14
C15 : 0	2.00	0.67	0.69	3.87
C16 : 0	24.15	5.01	14.76	37.77
C16 : 1	1.48	0.44	0.47	2.98
C17 : 0	0.70	0.23	0.36	2.05
C17 : 1	0.46	0.15	0.00	0.75
C18 : 0	3.60	2.25	0.68	9.50
C18 : 1n9	16.03	4.06	7.15	24.44
C18 : 2n6	3.21	1.26	0.63	6.30
C18 : 3n6	0.09	0.08	0.00	0.45
C18 : 3n3	0.36	0.14	0.00	0.76
C20 : 0	0.78	1.58	0.00	8.04
C20 : 1n9	0.058	0.040	0.00	0.140
C20 : 2	0.075	0.073	0.00	0.441
C20 : 3n6	0.028	0.027	0.00	0.088
C20 : 4n6	0.14	0.30	0.00	1.68
C21 : 0	0.21	0.10	0.00	0.40
C20 : 3n3	0.053	0.128	0.00	0.598
C20 : 5n3	0.025	0.026	0.00	0.080
C22 : 1n9	0.012	0.043	0.00	0.246
C22 : 2	0.062	0.081	0.00	0.371
C23 : 0	0.037	0.112	0.00	0.654
C24 : 0	0.020	0.030	0.00	0.124
C22 : 6n3	0.049	0.246	0.00	1.824
C24 : 1n9	0.005	0.009	0.00	0.037
SFA	70.52	5.23	53.84	83.86
MUFA	19.05	4.19	10.39	27.33
PUFA	4.09	1.45	1.55	9.36
n3	0.48	0.31	0.00	2.27
n6	3.44	1.34	0.87	8.08
n6 / n3	8.65	4.25	0.45	19.67

SD – standard deviation; SFA – saturated fatty acids; MUFA – monounsaturated fatty acids; PUFA – polyunsaturated fatty acids; n3 – total n3 fatty acids; n6 – total n6 fatty acids.

Analysis of the descriptive statistics referring to the content of fatty acids in sheep's milk and of the obtained values of individual groups of fatty acids (Table 4) showed increased values of the standard deviation for fatty acids present in milk fat in very small concentrations.

**Table 5:** Influence of lactation stages on fatty acid content and fatty acid groups in Lacaune ewes' milk (g 100 g${}^{-1}$ fat).

Parameter	Stage of lactation			SEM	P value
	I	II	III		
C4 : 0	3.55${}^{\mathrm{ab}}$	3.80${}^{a}$	2.97${}^{b}$	0.125	0.025
C6 : 0	2.83${}^{a}$	3.12${}^{a}$	2.10${}^{b}$	0.092	<0.001
C8 : 0	2.76${}^{a}$	2.94${}^{b}$	1.73${}^{b}$	0.094	<0.001
C10 : 0	10.20${}^{a}$	10.26${}^{a}$	5.86${}^{b}$	0.357	<0.001
C11 : 0	0.69${}^{a}$	0.72${}^{a}$	0.24${}^{b}$	0.031	<0.001
C12 : 0	7.12${}^{a}$	7.05${}^{a}$	4.85${}^{b}$	0.197	<0.001
C13 : 0	0.65${}^{a}$	0.69${}^{a}$	0.27${}^{b}$	0.029	<0.001
C14 : 0	11.99	12.16	12.31	0.171	0.742
C14 : 1	0.32${}^{b}$	0.29${}^{b}$	0.75${}^{a}$	0.027	<0.001
C15 : 0	2.19${}^{a}$	2.21${}^{a}$	1.53${}^{b}$	0.067	<0.001
C16 : 0	22.56${}^{b}$	22.37${}^{b}$	28.13${}^{a}$	0.499	<0.001
C16 : 1	1.42${}^{b}$	1.35${}^{b}$	1.68${}^{a}$	0.044	0.006
C17 : 0	0.72	0.75	0.63	0.022	0.105
C17 : 1	0.49${}^{a}$	0.50${}^{a}$	0.36${}^{b}$	0.015	<0.001
C18 : 0	2.74${}^{b}$	3.14${}^{b}$	5.22${}^{a}$	0.224	<0.001
C18 : 1n9	15.9	16.74	15.42	0.404	0.427
C18 : 2n6	3.62${}^{a}$	3.33${}^{a}$	2.54${}^{b}$	0.125	0.001
C18 : 3n6	0.12${}^{a}$	0.09${}^{\mathrm{ab}}$	0.07${}^{b}$	0.008	0.033
C18 : 3n3	0.34${}^{b}$	0.29${}^{b}$	0.44${}^{a}$	0.014	<0.001
C20 : 0	0.86	0.33	1.16	0.158	0.105
C20 : 1n9	0.063	0.052	0.058	0.0040	0.526
C20 : 2	0.07${}^{\mathrm{ab}}$	0.05${}^{b}$	0.10${}^{a}$	0.007	0.004
C20 : 3n6	0.024${}^{b}$	0.022${}^{b}$	0.040${}^{a}$	0.0027	0.008
C20 : 4n6	0.15	0.16	0.16	0.030	0.827
C21 : 0	0.02	0.20	0.20	0.010	0.595
C20 : 3n3	0.054	0.043	0.062	0.0128	0.842
C20 : 5n3	0.022${}^{\mathrm{ab}}$	0.018${}^{b}$	0.036${}^{a}$	0.0026	0.019
C22 : 1n9	0.012	0.014	0.010	0.0043	0.947
C22 : 2	0.070	0.035	0.084	0.0080	0.050
C23 : 0	0.027	0.023	0.066	0.0111	0.255
C24 : 0	0.020	0.016	0.024	0.0030	0.542
C22 : 6n3	0.007${}^{b}$	0.004${}^{b}$	0.152${}^{a}$	0.0245	0.022
C24 : 1n9	0.005	0.003	0.006	0.0009	0.403
SFA	70.76	70.41	70.34	0.545	0.938
MUFA	18.91	19.67	18.53	0.416	0.548
PUFA	4.48	4.00	3.69	0.144	0.075
n3	0.43${}^{b}$	0.35${}^{b}$	0.69${}^{a}$	0.031	<0.001
n6	3.89${}^{a}$	3.54${}^{\mathrm{ab}}$	2.76${}^{b}$	0.133	0.002
n6 / n3	9.76${}^{a}$	10.53${}^{a}$	5.27${}^{b}$	0.427	<0.001

SEM – standard error of the mean; ${}^{a,b}$ – means with different superscript letters differ significantly (p<0.05); SFA – saturated fatty acids; MUFA – monounsaturated fatty acids; PUFA – polyunsaturated fatty acids; n3 – total n3 fatty acids; n6 – total n6 fatty acids.

Analysis of the fatty acid profile in Lacaune sheep's milk sampled during various stages of lactation (Table 5) showed a significant decrease in concentrations of C4 : 0, C6 : 0, C11 : 0, C12 : 0, C13 : 0, C15 : 0, C17 : 1, C18 : 2n6, C18 : 3n6 and n6 as well as in the ratio n6 / n3. The opposite trend was noticed for concentrations of C10 : 0, C14 : 1, C16 : 0, C16 : 1, C18 : 0, C20 : 2, C18 : 3n3, C20 : 3n6, C20 : 5n3, C22 : 6n3 and n3. When compared to the early and middle lactation stages, concentrations of the above-mentioned fatty acids in Lacaune sheep's milk were lowest or highest in the late lactation stage, except for the concentrations of C4 : 0 and C8 : 0. Concentrations of C4 : 0 were significantly higher in the middle lactation stage compared to the late lactation stage. Concentrations of C8 : 0 were highest in the middle lactation stage, significantly higher when compared to the early lactation stage and significantly lower in the late lactation stage when compared to the early lactation stage. Concentrations of C18 : 3n6 and n6 were significantly lower in the late lactation stage than in the early lactation stage, while concentrations of C20 : 2 and C20 : 5n3 were significantly lower in the middle lactation stage than in the late lactation stage.

Tables 6 and 7 present the correlation coefficients determined between physical and chemical properties, hygienic indicators of milk and the daily yield of milk, and the content of fatty acids in Lacaune sheep's milk. There was a significant positive correlation determined between the following indicators in Lacaune sheep's milk produced during lactation: Fat : P, Fat : TS, Fat : SNF, Fat : Casein, Fat : TA, Protein : TS, Protein : SNF, Protein : Casein, Protein : TA, Lac : FFA, Lac : MY, TS : SNF, TS : Casein, TS : TA, SNF : Casein, SNF : TA, Casein : TA, Citric : Galact, Citric : Gluc, Density : SNF, FFA : FP and FP : MY. There were also significantly negative correlations determined for many indicators in Lacaune sheep's milk produced during lactation, such as Fat : Lac, Fat : FFA, Fat : FP, Fat : MY, Protein : Lac, Protein : FFA, Protein : FP, Lac : TS, Lac : Casein, Lac : TA, TS : FFA, TS : FP, TS : MY, SNF : FP, Casein : FFA, Casein : FP, Casein : TA, FP : TA, Galact : TA and Gluc : TA. A significant positive correlation was determined for the content of the following fatty acids and their n3 / n6 ratio: C12 : 0 to C14 : 0, SFA and the ratio n6 / n3; C14 : 0 to C16 : 0 and SFA; C16 : 0 to C18 : 0 and C18 : 3n3; C18 : 0 to polyunsaturated fatty acids (PUFA); C18 : 1 to C18 : 2n6, monounsaturated fatty acids (MUFA), PUFA and n6; C18 : 2 to MUFA, PUFA and n6; C18 : 3n3 to PUFA and n3; and MUFA to PUFA and n6. A significant negative correlation was determined for C12 : 0 to C16 : 0, C18 : 0, C18 : 1, C18 : n3 and n3; C14 : 0 to PUFA and n3; C16 : 0 to n6 and the ratio n6 / n3; C18 : 0 to C18 : 2, n6 and the ratio n6 / n3; C18 : 1 to SFA; C18 : 2 to SFA; C18 : 3n3 to SFA and the ratio n6 / n3; and SFA to MUFA, PUFA, n3, n6 and n6 / n3.

**Table 6:** Correlation coefficients between physical and chemical properties, hygienic indicators of milk and the daily yield of milk of Lacaune sheep.

	Fat	Protein	Lac	TS	SNF	Casein	Citric	Density	FFA	FP	Galact	Gluc	TA	Urea	logSCC	logCFU	MY
Fat	1.000	0.762 < 0.001	-0.523< 0.001	0.963 < 0.001	0.642 < 0.001	0.785 < 0.001	0.021 0.844	0.028 0.793	-0.435< 0.001	-0.492< 0.001	0.074 0.506	-0.131 0.264	0.539 < 0.001	0.117 0.271	0.081 0.447	0.318 0.002	-0.435< 0.001
Protein	0.762 < 0.001	1.000	-0.756< 0.001	0.847 < 0.001	0.702 < 0.001	0.998 < 0.001	-0.033 0.760	0.298 0.004	-0.694< 0.001	-0.440< 0.001	0.014 0.901	-0.098 0.406	0.675 < 0.001	-0.088 0.412	0.217 0.040	0.239 0.024	-0.580< 0.001
Lac	-0.523< 0.001	-0.756< 0.001	1.000	-0.479< 0.001	-0.109 0.3066	-0.746< 0.001	0.133 0.211	0.278 0.008	0.645 < 0.001	0.164 0.1218	-0.015 0.890	0.045 0.705	-0.412< 0.001	0.125 0.242	-0.221 0.036	-0.209 0.048	0.546 < 0.001
TS	0.963 < 0.001	0.847 < 0.001	-0.479< 0.001	1.000	0.792 < 0.001	0.863 < 0.001	0.087 0.414	0.261 0.013	-0.517< 0.001	-0.555< 0.001	0.082 0.464	-0.12 0.281	0.599 < 0.001	0.069 0.520	0.121 0.256	0.308 0.003	-0.460< 0.001
SNF	0.642 < 0.001	0.702 < 0.001	-0.109 0.307	0.792 < 0.001	1.000	0.725 < 0.001	0.038 0.722	0.657 < 0.001	-0.301 0.004	-0.480< 0.001	-0.148 0.183	-0.266 0.022	0.743 < 0.001	0.056 0.600	0.002 0.983	0.216 0.041	-0.287 0.007
Casein	0.785 < 0.001	0.998 < 0.001	-0.746< 0.001	0.863 < 0.001	0.725 < 0.001	1.000	-0.032 0.767	0.285 0.007	-0.664< 0.001	-0.444< 0.001	-0.004 0.973	-0.114 0.334	0.699 < 0.001	-0.059 0.583	0.196 0.064	0.255 0.015	-0.575< 0.001
Citric	0.021 0.844	-0.033 0.760	0.133 0.211	0.087 0.414	0.038 0.722	-0.032 0.767	1.000	0.038 0.720	0.173 0.107	0.132 0.216	0.534 < 0.001	0.539 < 0.001	-0.343 0.001	0.023 0.830	0.193 0.068	0.200 0.059	0.103 0.344
Density	0.028 0.793	0.298 0.004	0.278 0.008	0.261 0.013	0.657 < 0.001	0.285 0.007	0.038 0.720	1.000	-0.211 0.049	-0.295 0.005	-0.004 0.970	-0.044 0.712	0.328 0.002	-0.160 0.131	0.080 0.453	-0.070 0.510	-0.034 0.752
FFA	-0.435< 0.001	-0.694< 0.001	0.645 < 0.001	-0.517< 0.001	-0.301 0.004	-0.664< 0.001	0.173 0.107	-0.211 0.049	1.000	0.413 < 0.001	0.046 0.683	0.042 0.723	-0.339 0.001	0.167 0.120	-0.288 0.007	0.026 0.813	0.531 < 0.001
FP	-0.492< 0.001	-0.440< 0.001	0.164 0.122	-0.555< 0.001	-0.480< 0.001	-0.444< 0.001	0.132 0.216	-0.295 0.005	0.413 < 0.001	1.000	0.026 0.818	0.179 0.128	-0.411< 0.001	-0.391 0.001	-0.096 0.369	-0.209 0.048	0.479 < 0.001
Galact	0.074 0.506	0.014 0.901	-0.015 0.890	0.082 0.464	-0.148 0.183	-0.004 0.973	0.534 < 0.001	-0.004 0.970	0.046 0.683	0.026 0.818	1.000	0.638 < 0.001	-0.503< 0.001	0.040 0.722	0.103 0.356	0.042 0.705	-0.052 0.649
Gluc	-0.131 0.264	-0.098 0.406	0.045 0.705	-0.127 0.281	-0.2660.022	-0.114 0.334	0.539 < 0.001	-0.044 0.712	0.042 0.723	0.179 0.128	0.638 < 0.001	1.000	-0.546< 0.001	0.034 0.774	0.285 0.014	-0.124 0.292	0.090 0.454
TA	0.539 < 0.001	0.675 < 0.001	-0.412< 0.001	0.599 < 0.001	0.743 < 0.001	0.699 < 0.001	-0.343 0.001	0.328 0.002	-0.339 0.001	-0.411< 0.001	-0.503< 0.001	-0.546< 0.001	1.000	0.106 0.322	0.003 0.980	0.216 0.041	-0.369< 0.001
Urea	0.117 0.271	-0.088 0.412	0.125 0.242	0.069 0.520	0.056 0.601	-0.059 0.583	0.023 0.830	-0.160 0.131	0.167 0.120	-0.391< 0.001	0.040 0.722	0.034 0.774	0.106 0.322	1.000	-0.069 0.521	0.135 0.204	-0.192 0.074
logSCC	0.081 0.447	0.217 0.040	-0.221 0.036	0.121 0.256	0.002 0.983	0.196 0.064	0.193 0.068	0.080 0.453	-0.288 0.007	-0.096 0.369	0.103 0.356	0.285 0.014	0.003 0.980	-0.069 0.521	1.000	-0.083 0.435	-0.298 0.005
logCFU	0.318 0.002	0.239 0.024	-0.209 0.048	0.308 0.003	0.216 0.041	0.255 0.015	0.200 0.059	-0.070 0.510	0.026 0.813	-0.209 0.048	0.042 0.705	-0.124 0.292	0.216 0.041	0.135 0.204	-0.083 0.435	1.000	-0.231 0.031
MY	-0.435< 0.001	-0.580< 0.001	0.546 < 0.001	-0.460< 0.001	-0.287 0.007	-0.575< 0.001	0.103 0.344	-0.034 0.752	0.531 < 0.001	0.479 < 0.001	-0.052 0.6493	0.090 0.454	-0.369< 0.001	-0.192 0.074	-0.298 0.005	-0.231 0.031	1.000

TS – total solids; Lac – lactose; SNF – solid non-fat; FFA – free fatty acids; FP – freezing point; Galact – galactose; Gluc – glucose; TA – titratable acidity; SCC – somatic cell count; CFU – total number of microorganisms; MY – milk yield.

**Table 7:** Correlation coefficients between some of the content of fatty acids in Lacaune sheep's milk.

	C12 : 0	C14 : 0	C16 : 0	C18 : 0	C18 : 1	C18 : 2	C18 : 3	SFA	MUFA	PUFA	n3	n6	n6 / n3
C12 : 0		0.486 < 0.001	-0.488< 0.001	-0.650< 0.001	-0.284 0.0041	0.212 0.0337	-0.338< 0.001	0.437 < 0.001	-0.235 0.018	-0.062 0.536	-0.547< 0.001	0.119 0.2358	0.555 < 0.001
C14 : 0	0.486 < 0.001		0.351 < 0.001	-0.088 0.381	-0.203 0.042	-0.095 0.347	-0.041 0.681	0.605 < 0.001	-0.125 0.2130	-0.390< 0.001	-0.375< 0.001	-0.267 0.007	0.191 0.059
C16 : 0	-0.488< 0.001	0.351 < 0.001		0.625 < 0.001	0.144 0.151	-0.238 0.017	0.314 0.001	0.017 0.872	0.181 0.0697	-0.254 0.011	0.22 0.022	-0.323 0.001	-0.354< 0.001
C18 : 0	-0.650< 0.001	-0.088 0.381	0.625 < 0.001		0.204 0.041	-0.454< 0.001	0.265 0.008	0.010 0.925	0.148 0.139	-0.387< 0.001	0.283 0.004	-0.486< 0.001	-0.543< 0.001
C18 : 1	-0.284 0.004	-0.203 0.042	0.144 0.151	0.204 0.041		0.554 < 0.001	0.301 0.002	-0.851< 0.001	0.987 < 0.001	0.483 < 0.001	0.022 0.831	0.489 < 0.001	0.189 0.061
C18 : 2	0.212 0.034	-0.095 0.347	-0.238 0.017	-0.454< 0.001	0.554 < 0.001		0.250 0.012	-0.693< 0.001	0.577 < 0.001	0.898 < 0.001	-0.095 0.344	0.965 < 0.001	0.541 < 0.001
C18 : 3	-0.338 0.001	-0.041 0.681	0.314 0.001	0.265 0.008	0.301 0.002	0.250 0.011		-0.388< 0.001	0.302 0.002	0.344 < 0.001	0.477 < 0.001	0.230 0.021	-0.509< 0.001
SFA	0.437 < 0.001	0.605 < 0.001	0.017 0.872	0.010 0.925	-0.851< 0.001	-0.693< 0.001	-0.388< 0.001		-0.864< 0.001	-0.772< 0.001	-0.484< 0.001	-0.725< 0.001	-0.079 0.461
MUFA	-0.235 0.018	-0.125 0.213	0.181 0.070	0.148 0.139	0.987 < 0.001	0.577 < 0.001	0.302 0.002	-0.864< 0.001		0.488 < 0.001	-0.004 0.970	0.501 < 0.001	0.226 0.024
PUFA	-0.062 0.536	-0.389< 0.001	-0.254 0.011	-0.387< 0.001	0.483 < 0.001	0.898 < 0.001	0.344 < 0.001	-0.772< 0.001	0.488 < 0.001		0.261 0.008	0.962 < 0.001	0.277 0.006
n3	-0.547< 0.001	-0.375< 0.001	0.227 0.022	0.283 0.004	0.022 0.831	-0.095 0.344	0.477 < 0.001	-0.484< 0.001	-0.004 0.970	0.261 0.008		0.001 0.994	-0.620< 0.001
n6	0.119 0.236	-0.267 0.007	-0.323 0.001	-0.486< 0.001	0.489 < 0.001	0.965 < 0.001	0.230 0.021	-0.725< 0.001	0.501 < 0.001	0.962 < 0.001	0.001 0.994		0.459 < 0.001
n6 / n3	0.555 < 0.001	0.191 0.059	-0.354< 0.001	-0.543< 0.001	0.189 0.061	0.541 < 0.001	-0.509< 0.001	-0.079 0.461	0.226 0.024	0.277 0.006	-0.620< 0.001	0.459 < 0.001	

SFA – saturated fatty acids; MUFA – monounsaturated fatty acids; PUFA – polyunsaturated fatty acids; n3 – total n3 fatty acids; n6 – total n6 fatty acids.

## 4 Discussion

Assessment of the quality of sheep's milk is based on determination of physical and chemical properties, indicators of hygienic quality (SSC and CFU) and the fatty acid profile. Within this research, obtained results referring to the average content of milk fat (5.53 %), protein (5.90 %), lactose (4.51 %) and total dry matter (17.40 %) in milk of Lacaune sheep were similar to the values reported by authors from countries in Croatia's surroundings, with the exception of the lower content of milk fat determined in this research. In this research, the average morning quantity of milk from Lacaune sheep was 1.40 L, which indicated very good milk yield. In their experiment conducted on Lacaune sheep in Bulgaria, Panayotov et al. (2018a, b) determined a higher content of milk fat (7.6 % and 7.21 %) and a slightly higher or similar content of protein (7.09 % and 6.19 %) as well as lower or similar content of lactose (4.5 % and 3.67 %) than was the case in our research. The research conducted in Hungary by Libis-Márta et al. (2021) reported a higher fat content (7.78 %) and a similar protein content (5.78 %), while the daily milk yield was significantly lower than in our research (0.83 kg). Compared to our research, a slightly higher fat content (7.33 %) and a similar protein content (5.72 %) in milk of Lacaune sheep bred in France were determined by Pellegrini et al. (1997). They reported a lower morning milk yield of 948 mL. In the research conducted in Spain, Mehaba et al. (2021) reported average milk fat and protein contents of 6.81 % and 6.37 %, respectively, a similar lactose content (4.43 %) and a daily milk yield of 1.65 kg. In the research conducted on Lacaune sheep in Estonia, Tatar et al. (2022) also determined a higher content of milk fat (7.75 %), similar contents of protein (5.74 %) and lactose (4.76 %), and a similar morning milk yield (0.8–1.8 L). All the above-mentioned data confirm the fact that the increased milk yield affects decreased contents of basic milk ingredients (fat, protein and dry matter). This is caused by redirection of nutrients into body reserves within the preparation for the next reproductive cycle (conception or gravidity) (Mierlita et al., 2011). The above is supported by the established significant negative correlations between milk yield and basic chemical indicators in milk, such as fat, protein, dry matter and casein (Table 6; r=-0.435, -0.580, -0.460 and -0.575; P < 0.001), as well as by an established significantly positive correlation between lactose and FFA (Table 6; r=546 and 0.531; P < 0.001). Effects of intensive farming on Lacaune sheep in Bulgaria were researched by Nedeva et al. (2019). They reported that highly productive sheep had an average daily milk yield of 2.948 L, and sheep with low productivity gave 1.634 L of milk per day. In our research, the lower content of fat in milk of Lacaune sheep could be caused by a high milk yield and by feed rich in concentrated forage. The diet is one of the main environmental factors that influence the lipid content of milk. Angeles-Hernandez et al. (2020) determined that the composition of the ratio in sheep significantly affects milk fat and the content of fatty acids of milk. Compared to sheep fed a high-concentrate diet (concentrate content >40 % DM), those fed a high-forage diet (forage content >40 % DM) had an increased fat content in their milk. Unlike a high-forage diet, consumption of a high-concentrate diet can reduce biohydrogenation (Kucuk et al., 2001). In milk of Lacaune sheep bred in Bulgaria, Panayotov et al. (2018a) determined lower values for titratable acidity (16.00${}^{\circ }$ TH), as the expected value was 21–23${}^{\circ }$ TH, which would be comparable to results obtained in our research. The value for the density of milk was 1.032 g cm${}^{-3}$, which is expected and similar to the value determined in our research. In the EU, there is no limit set for the SCC in sheep's milk. However, Sevi et al. (1999) stated that sheep's milk is of a satisfactory hygienic and technological quality, at 70 000 cells per millilitre (6.85 log⁡10 cells per millilitre), while Kasapidou et al. (2021) investigated Greek sheep breeds (Karagouniko, Maoutsiko, Artas and Chios) and reported an average SSC in milk produced in intensive sheep farming of 3.271log⁡10 cells per millilitre. As stated by Panayotov et al. (2018a), milk produced by Lacaune sheep in Bulgaria had 754 000 microorganisms (CFU, g mL${}^{-1}$) and an SSC of 147 000 g mL${}^{-1}$. Gonzalo et al. (2000) considered sheep's milk with an SCC of lower than 500 000 (5.70 log⁡10 cells per millilitre) to be of good quality. The average values of the SCC in Lacaune sheep's milk obtained in this research were determined in accordance with the above-mentioned values.

As lactation progressed, it affected significant increases in the content of most of the physical and chemical indicators in milk of Lacaune sheep, which was also associated with the decrease in daily milk yield per ewe up to the end of lactation. Only the content of lactose in milk significantly decreased as lactation progressed, which was expected. No significant changes were observed in the indicators of the hygienic quality of milk (SCC and CFU). Sevi et al. (1999) linked the insignificant increase in SCC in sheep's milk to a reduced milk yield during lactation or to functional alternation of the mammary gland tissue caused by the shorter survival of epithelia cells, which are supplied with fewer nutrients at the end of lactation. Such an occurrence can also be connected to a significant negative correlation between MY and SCC (Table 6; r=-0.98; P=0.005). In their research into Lacaune sheep in the Czech Republic, Kuchtik et al. (2017) published similar observations for most of the physical and chemical properties and for indicators of the hygienic quality of milk during the late lactation stage. They determined a higher content of fat and dry matter in Lacaune sheep's milk at the beginning and end of lactation (6.25 % and 7.69 % as well as 17.61 % and 19.48 %, respectively) and a significantly lower daily milk yield, which was 1.07 L on the 57th day of lactation, and towards the end of lactation, on the 197th day, it was 0.77 L. They also reported variations in SCC that were similar to our research.

Results similar to ours were also reported by Brito et al. (2006) for most physical and chemical indicators in the milk of Lacaune sheep bred in Brazil. The titratable acidity (TA) values in Lacaune sheep's milk can be associated with the increase in protein content in milk during lactation, which was confirmed by a strong positive correlation (Table 6; r=0.675; P < 0.001). Similar conclusions were published by Novotna et al. (2009). The increase in urea in the milk of Lacaune sheep towards the late stage of lactation can be compared to values measured in Krk's sheep by Bendelja et al. (2009) and in cows as reported by Carlsson et al. (1995). The above-mentioned facts can be connected to lower requirements for protein to be taken in through feed because of significant decreases in milk yield at the end of lactation. Many significant positive and negative correlations between physical, chemical and hygienic quality indicators of milk of Lacaune sheep, together with the milk yield, showed significant interdependence of these indicators during lactation and are in accordance with the results obtained in other studies performed on Lacaune sheep (Kuchtik et al., 2017; Tatar et al., 2022). Pellegrini et al. (1997) also confirmed an increase in most of the physical and chemical indicators in milk of Lacaune sheep bred in France as well as a significant decrease in the morning milk yield, which was 1374 mL in the period from 48 to 55 d of lactation and 513 mL in the period from 174 to 192 d of lactation. In their research conducted in Spain, Jimenez et al. (2020) determined that Lacaune sheep had an average daily milk production of 1.07 L over a lactation period of 160 d. Molina et al. (2001) also confirmed a significant decrease in the amount of milk as the lactation of Lacaune sheep approached its end. Nedeva et al. (2019) researched Lacaune sheep in Bulgaria and determined that the daily milk yield in highly productive sheep decreased from 3.122 to 2.838 L as lactation progressed, and in low-productivity sheep it decreased from 1.993 to 1.367 L, which indicated good adaptability. Oravcová et al. (2006) reached similar conclusions in their research into Lacaune sheep in Slovakia. They determined significant differences in the daily milk yield of 1.5–1.9 kg during the peak lactation period, and at the end of lactation it dropped to 0.64–0.69 kg. As emphasized by Alba et al. (2020), after implementation of the strict selection programme by the French governmental agency, recent research showed that Lacaune sheep in France give 400–500 kg of milk during lactation, which makes them one of the most highly productive sheep breeds in the world (Li et al., 2022).

Sánchez et al. (2010) determined that Spanish Churra sheep's milk contained the most SFA (66 %), followed by MUFA (28 %) and then PUFA (6 %). In our research, milk of Lacaune sheep had a higher content of SFA (70.52 %) and lower contents of MUFA (19.05 %) and PUFA 4.09 %. The most represented fatty acids in milk of lactating Lacaune sheep were C14 : 0, C16 : 0 and C18 : 1n9, and the most dominant monounsaturated and polyunsaturated fatty acids were oleic and linoleic fatty acids. Palmitic acid (C16 : 0) was the most represented one in milk of Lacaune sheep. Similar conclusions were reached by Kasapidou et al. (2021) while investigating the milk of crossbred dairy sheep in Greece and by Gatzias et al. (2018) in their study on Greek sheep breeds (Karagouniko, Maoutsiko, Artas and Chios). Our research resulted in high variations of individual fatty acids, especially long-chained fatty acids (Table 4). The above-mentioned ones led to significantly high correlations between positive or negative trends for most of the calculated fatty acids and their groups (SFA, MUFA and PUFA; Table 7). Similar conclusions were drawn by Conte et al. (2022) while researching the milk of Italian sheep breeds. Feeding regime has a more significant influence on the content of fatty acids in sheep's milk than genotype or breed (Tsiplakou et al., 2008). Although Conte et al. (2022) pointed out that sheep breeds reared in the same farming system (Comisana, Massese and Sarda) had a significant influence on the factor that affected FA composition in milk, they also suggested the possibility of genetic improvement. Over the lactation period, the sum of short-chain fatty acids (C4 : 0–C6 : 0) and the sum of medium-chain fatty acids (C8 : 0–C13 : 0) in Lacaune sheep's milk were reduced by 25.8 % and 65.4%, respectively. Sinanoglou et al. (2015) also published similar conclusions in their study into effects of the lactation stages on the content of fatty acids in milk produced by Greek sheep breeds (Karagouniko and Chios). The lactation stage of Lacaune sheep affected significant increases in palmitic acid (C16 : 0) and stearic acid (C18 : 0) and a decrease in lauric acid (C12 : 0) in late lactation when compared to early lactation. Lactation stages of Chios and Karagouniko sheep also exhibited similar influences on the content of lauric acid in milk, while milk of Chios sheep had the highest content of stearic acid at the end of lactation (Sinanoglou et al., 2015). Inostroza et al. (2020) researched the influence of the lactation stage on the variation in milk composition and the fatty acid profile in milk of Araucana Creole ewes kept in pasture-based systems and determined significant differences, which they connected with the balance between body fat mobilization and de novo synthesis of fatty acids in the mammary glands. In early lactation in sheep, there is a negative energy balance present, and the fat and long-chain fatty acid content in milk increase due to the uptake of non-esterified fatty acids derived from body fat mobilization (Pulina et al., 2006). Fernandez and Rodriguez (2012) pointed out that short- to medium-chain fatty acids (from C4 : 0 to C15 : 0) were derived from de novo synthesis in the mammary glands, and the long-chain fatty acids (from C17 : 0 to C26 : 0) were derived from the diet. Increased energy of highly productive Lacaune dairy sheep fed by high-quality forage ad libitum did not improve milk yield or change milk composition but increased sheep body weight and body condition score (Hassoun et al., 2021). Cabiddu et al. (2005) stated that sheep grazing on Mediterranean pastures produced milk enriched with fatty acids (n3 PUFA) which are beneficial to human health. In their study on Massese sheep fed diets with different shares of voluminous forage and concentrate, Martini et al. (2010) observed that the increase in voluminous feed led to an increase in the fat content of milk, to a decrease in the percentage of some medium-chain fatty acids, and to an increase in the percentage of monounsaturated and polyunsaturated fatty acids in milk, thus indicating a need to use larger amounts of forage in sheep diets.

## 5 Conclusion

Average values obtained in this research for physical and chemical properties, indicators of the hygienic quality and the fatty acid profile of sheep's milk were comparable to the results of previous studies performed on Lacaune sheep, except for the lower content of milk fat, which could be associated with a larger quantity of milk. The influence of lactation stages was significant for most of the physical and chemical properties of Lacaune sheep's milk, as there was a significant increase in their content observed as lactation progressed, yet the opposite trend was determined for the content of lactose. No significant changes were confirmed for the indicators of the hygienic quality of Lacaune sheep's milk, depending on the stage of lactation. The content of most of the fatty acids in milk was reduced as lactation approached its end, while the contents of the fatty acids C14 : 1, C16 : 0, C10 : 1, C18 : 0, C18 : 3n3, C20 : 2, C20 : 5n3 and C22 : 6n3 increased. The sum of n3 fatty acids and the ratio of n6 / n3 were also lower, along with the progress of lactation. Comprehensive analysis of sheep's milk ingredients proved Lacaune sheep to be highly adaptable, with the potential for expansion of the breed to continental Croatia, where sheep can be provided with quality pastures.

## Data Availability

The data are available from the corresponding author upon reasonable request.
